# Complex I deficiency in m.3243A>G fibroblasts is alleviated by reducing NADH accumulation

**DOI:** 10.3389/fphys.2023.1164287

**Published:** 2023-08-15

**Authors:** Tongling Liufu, Haiyan Yu, Jiaxi Yu, Meng Yu, Yue Tian, Yichun Ou, Jianwen Deng, Guogang Xing, Zhaoxia Wang

**Affiliations:** ^1^ Department of Neurology, Peking University First Hospital, Beijing, China; ^2^ Department of Clinical Laboratory, Peking University First Hospital, Beijing, China; ^3^ Neuroscience Research Institute, Peking University, Beijing, China; ^4^ Beijing Key Laboratory of Neurovascular Disease Discovery, Beijing, China

**Keywords:** complex I, m.3243 A>G, mitochondrial disease, mitoLbNOX, NADH, nr

## Abstract

**Introduction:** Mitochondrial disease is a spectrum of debilitating disorders caused by mutations in the mitochondrial DNA (mtDNA) or nuclear DNA that compromises the respiratory chain. Mitochondrial 3243A>G (m.3243 A>G) is the most common mutation showing great heterogeneity in phenotype. Previous studies have indicated that NADH: ubiquinone oxidoreductase (complex I) deficiency accompanied by a decreased nicotinamide adenine dinucleotide (NAD^+^)/reduced NAD^+^ (NADH) ratio may play a pivotal role in the pathogenesis of m.3243A>G mutation.

**Methods:** To evaluate the potential effects of strategies targeting the imbalanced NAD^+^/NADH ratio in m.3243A>G mutation, we treated fibroblasts derived from patients with the m.3243 A>G mutation using nicotinamide riboside (NR) or mitochondria-targeted H_2_O-forming NADH oxidase (mitoLbNOX).

**Results:** M.3243 A>G fibroblasts showed a significant reduction in complex I core subunit 6, complex I enzymatic activity, complex I-dependent oxygen consumption rate (OCR), and adenosine triphosphate (ATP) production compared to the controls. The NAD^+^/NADH ratio was also significantly reduced in m.3243 A>G fibroblasts, and, using fluorescence lifetime imaging microscopy, we also found that the NADH level was elevated in m.3243 A>G fibroblasts. After NR treatment, the NAD^+^/NADH ratio, complex I-dependent OCR, and ATP levels increased, whereas NADH levels remained unchanged. More excitingly, after treatment with mitoLbNOX, the NAD^+^/NADH ratio, complex I-independent OCR, and ATP levels increased more pronouncedly compared with the NR treatment group, accompanied by significantly reduced NADH levels.

**Discussion:** The present study suggests that compared with repletion of NAD^+^ alone, the combination of this therapeutic modality with alleviation of NADH overload may amplify the treatment effect of restoring NAD^+^/NADH balance in m.3243A>G fibroblasts.

## 1 Introduction

Mutations in mitochondrial DNA (mtDNA) or nuclear DNA (nDNA) lead to primary mitochondrial diseases, characterized by respiratory chain dysfunction (Ng et al., 2021). Mitochondrial 3243A>G (m.3243 A>G) is the most common mutation presenting with a wide range of phenotypes including mitochondrial encephalomyopathy with lactic acidosis and stroke-like episodes (MELAS), maternally-inherited diabetes and deafness (MIDD), myopathy, cardiomyopathy, etc (Thompson et al., 2020). Two observational studies concluded that MELAS has high mortality and morbidity, with stroke-like episodes being the most frequent symptom (Yatsuga et al., 2012; Zhang et al., 2018). In a handful of palliative therapies, L-arginine is a canonical drug used to prevent stroke-like episodes (Koga et al., 2018). Another interesting finding of an open-label clinical trial was that taurine may reduce the relapse rate of stroke-like episodes (Ohsawa et al., 2019). Some studies suggest that glucose deprivation may serve as a potential treatment strategy for m.3243A>G related diseases in the future (Frey et al., 2017; Geffroy et al., 2018). As m.3243A>G related diseases remains untreatable and devastating, the development of new drugs and treatment strategies is urgently needed.

NADH: ubiquinone oxidoreductase (complex I) deficiency is a prevalent feature in biochemical and pathological studies of m.3243A>G associated MELAS (Goto et al., 1992; Alston et al., 2017; Lu et al., 2020). Complex I is the major enzyme that regenerates NAD^+^ for continued catabolism and the function of NAD^+^-dependent enzymes such as sirtuins (Cantó et al., 2015). Depletion of NAD^+^ has been studied for the treatment of other subtypes of mitochondrial diseases. Patients with adult-onset mitochondrial myopathy showed improved muscle performance after niacin treatment (Pirinen et al., 2020). While beyond NAD^+^ depletion, recent reports showed that NADH accumulated in cells when complex I was impaired (Birsoy et al., 2015; Sullivan et al., 2015). NADH overload is associated with reactive oxygen species production, blockade of tricarboxylic acid (TCA) turning, impairment of *de novo* aspartate synthesis, and transcriptional regulation (Yoo et al., 2008; Mullen et al., 2011; Wise et al., 2011; Gameiro et al., 2013; Vinogradov and Grivennikova, 2016; Levine et al., 2021). Using mouse models of Leigh syndrome, several promising strategies have been developed to reduce excess NADH, including the expression of exogenous NADH dehydrogenase, offering alternative electron acceptors, and inhibiting mitochondrial serine catabolism (Bai et al., 2001; Titov et al., 2016; Lozoya et al., 2018; McElroy et al., 2020; Yang et al., 2020).

Both clinical studies and cellular models have indicated the NAD^+^/NADH ratio is decreased in MELAS (Frey et al., 2017; Sharma et al., 2021). Previous studies have mainly focused on approaches to increase the cellular levels of NAD^+^ (Majamaa et al., 1996; Zhang et al., 2013; Jeong et al., 2014; Frey et al., 2017; Seo et al., 2018). The role of NADH accumulation and its potential treatment efficacy in MELAS warrant further exploration. Here, we used fibroblasts from patients with m.3243 A>G mutation to further investigate the role of an imbalanced NAD^+^/NADH ratio in m.3243A>G mutation. After treatment with nicotinamide riboside (NR), the NAD^+^/NADH ratio, complex I-dependent oxygen consumption rate (OCR), and adenosine triphosphate (ATP) levels were elevated, while NADH levels remained unchanged. It is noteworthy that mitochondria-targeted H_2_O-forming NADH oxidase (mitoLbNOX), an NADH oxidase from *Lactobacillus brevis*, significantly reduced the excess load of NADH and further improved oxidative metabolism, including the NAD^+^/NADH ratio, OCR, and ATP levels in m.3243A>G fibroblasts. Therefore, our study revealed that, compared with the sole repletion of NAD^+^, combined with alleviation of NADH overload, may amplify the treatment effect of restoring the NAD^+^/NADH balance in m.3243A>G fibroblasts.

## 2 Material and methods

### 2.1 Ethics statement

The patients provided written informed consent for basic information publication and skin biopsy for fibroblasts culture. The experiments using human cells were approved by the Ethics Committee of Peking University First Hospital (Ethic code: 2021 [061]).

### 2.2 Cell culture

Skin biopsy specimens were obtained from patients with m.3243A>G mutation and healthy control individuals. The basic patient information is shown in Table 1. Human fibroblasts were cultured in Dulbecco’s modified Eagle’s medium (DMEM; BI, Cat# 06-1055-57-1A) supplemented with 20% fetal bovine serum (FBS; BI, Cat# 04-001-1A) and 100 IU/mL penicillin/streptomycin (BI; Cat# 03-031-1B). Due to proliferation defects and growth discrepancies, not all fibroblast lines were included in all experiments. Control fibroblasts at passages 1–10 and MELAS fibroblasts at passages 4–12 were used for experiments.

**TABLE 1:** Basic information of the four patients included in this research. Pt: Patient.

No.	ID	Sex	Age of onset	Disease duration	Clinical diagnosis	Mutation
1	Pt1	Female	42 years old	1 year	MELAS	m.3243 A>G
2	Pt2	Female	11 years and 3 months old	2 years	MELAS	m.3243 A>G
3	Pt3	Female	38 years old	20 years	Mitochondrial myopathy	m.3243 A>G
4	Pt4	Female	16 years and 5 months old	2 months	MELAS	m.3243 A>G

### 2.3 Construction of MitoLbNOX adenovirus

The custom adenovirus was obtained from WZ Biosciences. The plasmid containing the mitoLbNOX nucleotide sequence reported by Titov et al. (Titov et al., 2016) was acquired from Addgene (Vydt et al., 2007). The mitoLbNOX-coding sequence was excised from pUC57 and directly ligated into the pADM-CMV-C-FH-mCMV-copGFP vector (WZ Biosciences). The MitoLbNOX adenovirus co-expresses green fluorescent protein (GFP) and mitoLbNOX, driven by the cytomegalovirus (CMV) promoter. An adenovirus with GFP driven by the CMV promoter was used as the control (WZ Bioscience).

### 2.4 NR treatment and adenovirus infection

Fibroblasts were treated with NR or adenovirus. For the NR treatment, 0.5, 1, or 3 mM NR (Selleck, Cat# S2935) was added to the fibroblast medium, and the cells were collected after 7 days for further analysis. For adenovirus infection, fibroblasts were infected with 3.5 × 10^10^ pfu/mL adenovirus for 24 h, and the cells were then collected.

### 2.5 Transmission electron microscopy

Fibroblasts were fixed with 2.5% glutaraldehyde (Sigma) at 4°C overnight. The samples were then dehydrated using a series of graded ethanol solutions and embedded in resin. After embedding, 70 μm sections were prepared with a Leica EM UC6/FC6 ultramicrotome. Images were obtained using a Phillips 410 transmission electron microscope. The size of the mitochondria was measured from the photographs of each fibroblast line, by calculating the average area of 80–100 mitochondria, as described previously (Deng et al., 2018).

### 2.6 Restriction fragment length polymorphism-Polymerase chain reaction and Sanger sequencing

Total DNA was extracted from the fibroblasts using a TIANamp Genomic DNA Kit (Tiangen). A segment of mtDNA was amplified by PCR using the primers: forward, 5′cct​cgg​agc​aga​acc​caa​cct 3′; reverse, 5′cga​agg​gtt​gta​gta​gcc​cgt 3′. For m.3243 A>G, the PCR products were digested with ApaI into two fragments. The fragments were separated on 2% agarose gel and stained with ethidium bromide (E43590-1g; Acmec). The signal in the gel was detected using PhosphorImager. The ImageJ software was used to analyze the signal intensities of the bands. The mutation was confirmed by Sanger sequencing.

### 2.7 Western blotting

Fibroblasts were lysed with RIPA lysis buffer (Beyotime, Cat# P0013D). Lysates were analyzed by Western blotting using antibodies against complex I core subunit 6 (ND6) (BIOSS, Cat# bs-3955R), TOM20 (Proteintech, Cat# 11802-1-AP), and Flag (CWBIO, Cat# CW0287S). Band intensity was measured using ImageJ software.

### 2.8 Blue-native PAGE (in-gel activity)

Briefly, mitoplasts, prepared from fibroblasts by treatment with 1.2 mg digitonin per mg of protein, and samples containing 100 μg mitochondrial protein were separated on NativePAGE 4%–16% Bis-Tris gels (Thermo Fisher Scientific, Cat# BN1002BOX). For the assessment of complex I activity, the gel was transferred to a solution of 2 mM Tris-HCl pH 7.4 (Sigma-Aldrich, Cat# 10812846001) containing 0.1 mg/mL nicotinamide adenine dinucleotide (Sigma-Aldrich, Cat# 481913-500 MG) and 0.25 mg/mL nitro blue tetrazolium (Sigma-Aldrich, Cat# N5514-10TAB) at 37°C with mild agitation. Banding began to develop within 2 h with an optimal band visualization at >24 h. 10 μg mitochondrial protein were analyzed by Western blot with antibody against TOM20 (as loading control).

### 2.9 Spectrophotometric assays of complex IV activity

The complex IV activity was measured using a complex IV activity assay kit (Solarbio, Cat# BC0940), according to the manufacturer’s instructions.

### 2.10 Oxygen consumption rate detection

OCR was measured using high-resolution respirometry (Oroboros Oxygraph-2K, Austria) in intact cells. The integrated software (Datlab 4.2) presents respiration as oxygen flux; pmol O_2_ per 10^6^ cells per second. OCR were measured under basal condition and after sequential injections of oligomycin (Omy; 1 μM; Sigma Aldrich, Cat# 75351), carbonyl cyanide m-chlorophenylhydrazone (CCCP; 2 μM; Sigma Aldrich, Cat# C2759), rotenone (Rot; 1 μM; Sigma Aldrich, Cat# 557368) and antimycin A (Ama; 1 μM; Sigma Aldrich, Cat# A8674).

### 2.11 Measurement of NAD^+^/NADH ratio (WST8 test)

The NAD^+^/NADH ratio was measured using an NAD^+^/NADH assay kit (Beyotime, Cat# S0175), according to the manufacturer’s instructions. Fibroblasts were collected and lysed by 800 μL lysis buffer for 15 min at room temperature. After centrifuged at 15,000 rpm for 3 min at 4°C, 60 μL supernatants were used to measure NAD^+^ and NADH. The supernatants were mixed with detection reagents in a 96 well plate (NEST, Cat# 703001). The signals were recorded at 450 nm after 45 min of incubation at room temperature.

### 2.12 FLIM of NADH (NADH FLIM)

NADH FLIM was performed on fibroblasts grown in glass-bottomed dishes (NEST, Cat# 801001) in fibroblast medium. Fibroblasts were imaged using a Leica SP8 FALCON with a DIVE laser scanning microscope. An oil-immersion objective of ×63/1.4NA was used for image acquisition. The NADH FLIM signal was collected using 750 nm 2-photon excitation (Spectra-Physics InSight X3 tunable laser, 0.8 mW average power) and 408–479 nm emission. Fluorescence was monitored using a multi-detector approach (Fast FLIM). Five images were obtained for each sample. Five to six fibroblasts with clear boundaries and the correct relative positions were selected from each image. The region of interest of each fibroblast was manually extracted to calculate free and protein-bound NADH. Proper fitting of the lifetime curve was evaluated using χ^2^, and the mean lifetime (τmean) was calculated.

### 2.13 Measurement of adenosine triphosphate level

Cellular ATP levels were measured using the Celltiter-Glo Luminescent Cell Viability Assay (Promega, Cat# G7570), according to the manufacturer’s instructions. Luminescence was measured using a MicroPlate Reader and the values were normalized to the cell number.

### 2.14 Statistical analysis

Differences between groups were evaluated using the unpaired two-tailed Student’s t-test, Mann-Whitney test (two groups), or one-way ANOVA with the Bonferroni *post hoc* test (multiple groups). Asterisks (*, **, ***, and ****) indicate significant differences (*p* < 0.05, *p* < 0.01, *p* < 0.001, and *p* < 0.0001, respectively).

## 3 Results

### 3.1 Expression of ND6 and activity of complex I were impaired in m.3243A>G fibroblasts

**FIGURE 1:**
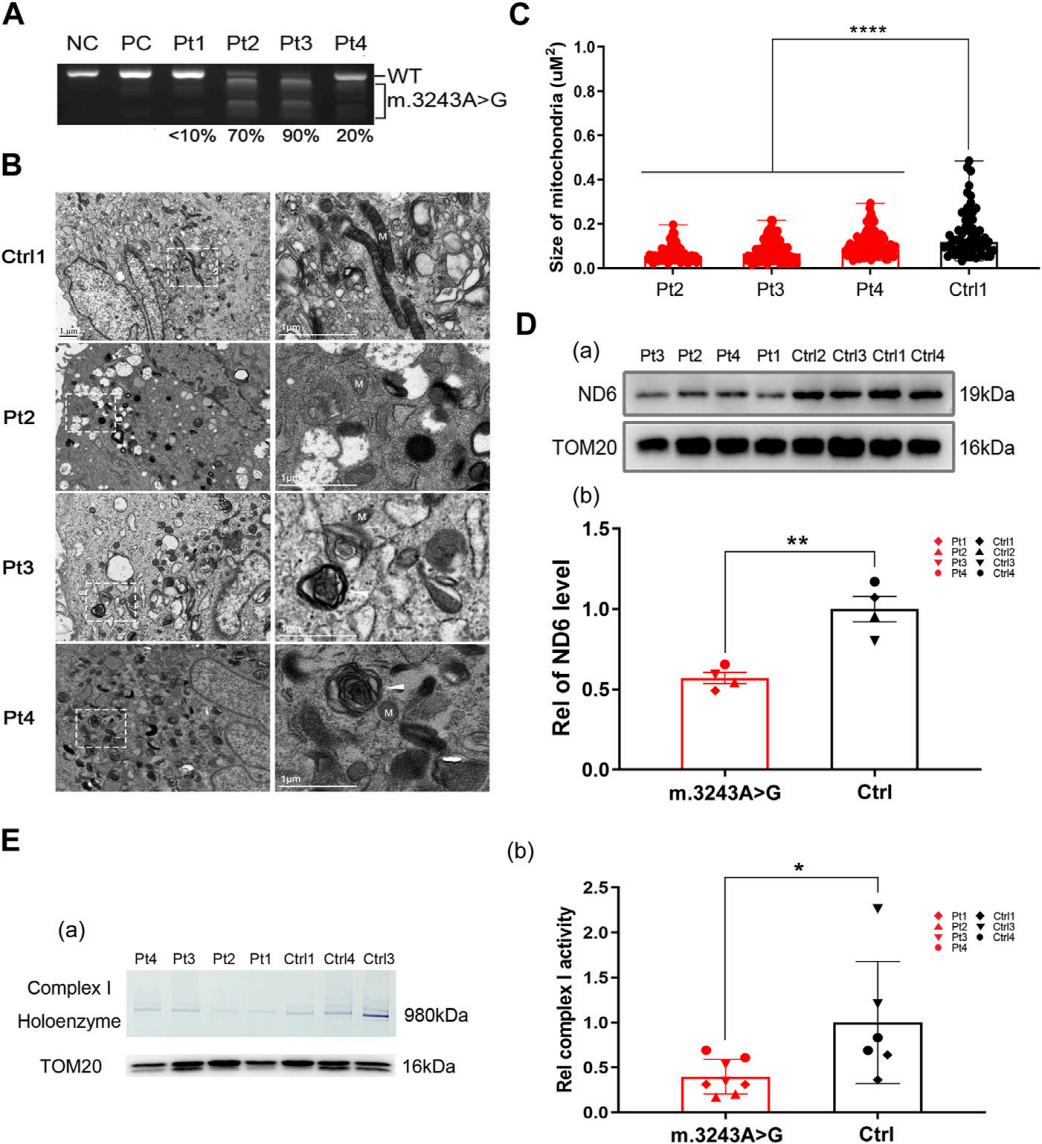
The decreased expression of ND6 may account for the deficiency in complex I observed in the m.3243A>G fibroblasts. **(A)** The m.3243A>G mutation rate of m.3243A>G fibroblasts was determined by RFLP analysis compared to negative control and positive control. **(B)** Mitochondria (M) in m.3243A>G and control fibroblasts were visualized by transmission electron microscopy. Arrowheads indicate myelin figures. **(C)** The size of the mitochondria in m.3243A>G and control fibroblasts was measured as described in the materials and methods section (number of mitochondria counted: m.3243A>G n = 74–109, Control n = 63; data were analyzed using a one-way ANOVA with Bonferroni *post hoc* test; error bars represent mean ± SEM; ****p < 0.0001). **(D)** Western blotting **(A)** and quantification **(B)** for relative expression level of ND6 protein in m.3243A>G and control fibroblasts (m.3243A>G n = 4, Control n = 4; data were analyzed using a Student’s t-test; error bars represent mean ± SEM; **p < 0.01). **(E)** BN PAGE (In-gel activity) analysis **(A)** and quantification **(B)** of the complex I activity in m.3243A>G and control fibroblasts using SDS-PAGE analysis of TOM20 protein as mitochondrial loading marker (m.3243A>G n = 4, Control n = 3; data were analyzed using a Mann Whitney U test; error bars represent mean ± SEM; *p < 0.05). ANOVA, analysis of variance; BN-PAGE, blue native polyacrylamide gel electrophoresis; RFLP, Restriction fragment length polymorphism; SDS-PAGE, sodium dodecyl sulfate polyacrylamide gel electrophoresis; SEM, standard error of the mean.

To investigate the effect of m.3243 A>G mutation, we collected skin biopsy specimens from four patients (3 MELAS and 1 mitochondrial myopathy) with m.3243 A>G mutation for primary fibroblast culture. The m. 3243 A>G heteroplasmy levels in patient-derived fibroblasts ranged from less than 10%–90% (Figure 1A). The m.3243 A>G mutation in each fibroblast line was further confirmed by Sanger sequencing (Supplementary Figure S1A). Fibroblasts with high levels (>60%) of m.3243 A>G heteroplasmy from patients 2 and 3 showed stable heteroplasmy levels during the experiments (Supplementary Figure S1B). To verify mitochondrial abnormalities in these fibroblast cell models, we performed electron microscopy on control and m.3243 A>G fibroblasts. Small electron-lucent mitochondria and myelin figures were detected more frequently in m.3243A>G fibroblasts than in control fibroblasts (Figure 1B). The mitochondria in m.3243A>G fibroblasts were smaller than those in the control fibroblasts (Figure 1C). The level of ND6 protein in m.3243A>G fibroblasts was approximately 60% of that in control fibroblasts (Figure 1D), indicating an apparent defect in ND6 expression. BN-PAGE (blue native polyacrylamide gel electrophoresis) and in-gel activity analysis revealed that complex I enzymatic activity in m.3243A>G fibroblasts was reduced to 41% of that in control fibroblasts (Figure 1E). There was no significant difference in complex IV activity between the m.3243A>G and control fibroblasts as revealed by the spectrophotometric assays (Supplementary Figure S1C).

### 3.2 NAD^+^ depletion and NADH accumulation coexisted in m.3243A>G fibroblasts

**FIGURE 2:**
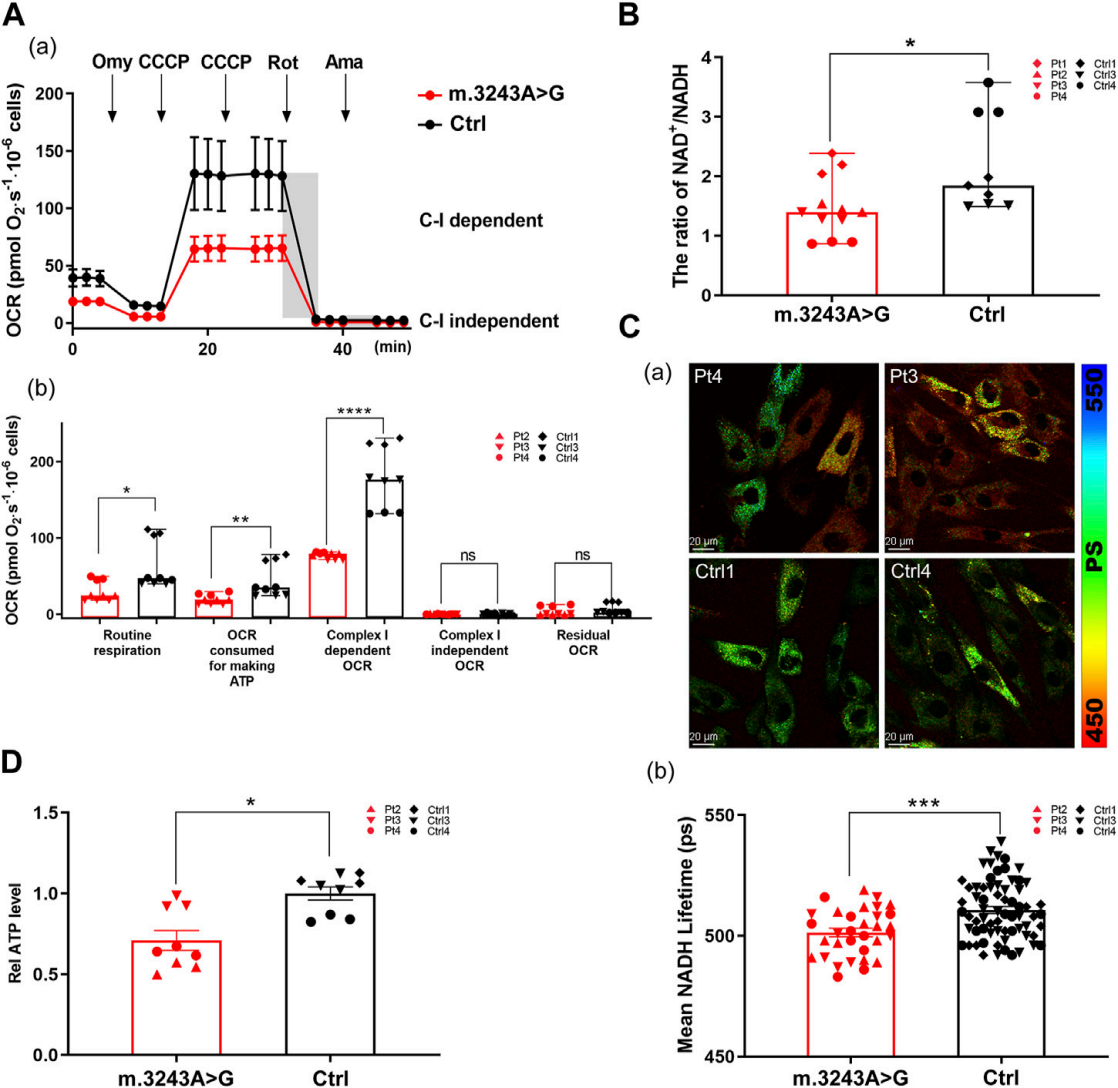
Imbalanced NAD^+^/NADH ratio and reduced ATP level were seen in m.3243A>G fibroblasts when complex I-dependent respiration is impaired. **(A)** Oxygraphic measurements **(A)** and analysis **(B)** in m.3243A>G and control fibroblasts (m.3243A>G n = 9, Control n = 9; data were analyzed using a Mann Whitney U test; error bars represent mean ± SEM; *p < 0.05, **p < 0.01, ****p < 0.0001, ns: not significant). **(B)** Relative NAD^+^/NADH ratio of m.3243A>G and control fibroblasts (m.3243A>G n = 12, Control n = 9; data were analyzed using a Mann Whitney U test; error bars represent mean ± SEM; *p < 0.05). **(C)** Pseudocolored images **(A)** and quantification **(B)** of NADH lifetime in m.3243A>G and control fibroblasts (number of fibroblasts counted: m.3243A>G n = 32, Control n = 68; data were analyzed using a Student’s t-test; error bars represent mean ± SEM; ***p < 0.001). **(D)** Relative ATP levels of m.3243A>G and control fibroblasts (m.3243A>G n = 9, Control n = 9; data were analyzed using a Student’s t-test; error bars represent mean ± SEM; *p < 0.05). ATP, adenosine triphosphate; NAD, nicotinamide adenine dinucleotide; NADH, nicotinamide adenine dinucleotide **+** hydrogen; SEM, standard error of the mean.

To evaluate oxidative phosphorylation defects, we analyzed the OCR in m.3243A>G and control fibroblasts. The routine respiration and OCR consumed for ATP production in m.3243A>G fibroblasts were significantly decreased by 53% and 54%, respectively, compared with control fibroblasts (Figure 2A; Supplementary Figure S2A), suggesting that the m.3243 A>G mutation impairs the basal respiratory capacity and ATP produced by respiratory chain. We defined the reduced portion of OCR after the addition of Rot as complex I-dependent OCR (Liu et al., 2021). The complex I-dependent OCR of m.3243A>G fibroblasts was reduced by more than 50% compared to that of control fibroblasts (Figure 2A), indicating complex I dysfunction. The substrate of succinate, ubiquinone oxidoreductase (complex II), is not produced in living cells after inhibiting complex I with Rot because of the blockage of the TCA cycle (Dienel, 2019). Thus, complex I-independent OCR approached zero in all fibroblasts (Figure 2A). There was no significant difference in the residual OCR between the m.3243A>G and control fibroblasts (Figure 2A), indicating a stable background state during the experiments. We further analyzed the NAD^+^/NADH ratio and used FLIM to separately evaluate NADH levels. In FLIM, decreased NADH lifetime is correlated with increased free NADH (Kopinski et al., 2019). We found that the NAD^+^/NADH ratio in m.3243A>G fibroblasts was 0.6-fold of that in control fibroblasts (Figure 2B), and that the NADH lifetime of m.3243A>G fibroblasts was lower than that of control fibroblasts (Figure 2C). ATP production in m.3243A>G fibroblasts was 20% lower than that in control fibroblasts (Figure 2D).

### 3.3 NAD^+^ repletion improved oxidative metabolism in m.3243A>G fibroblasts

**FIGURE 3:**
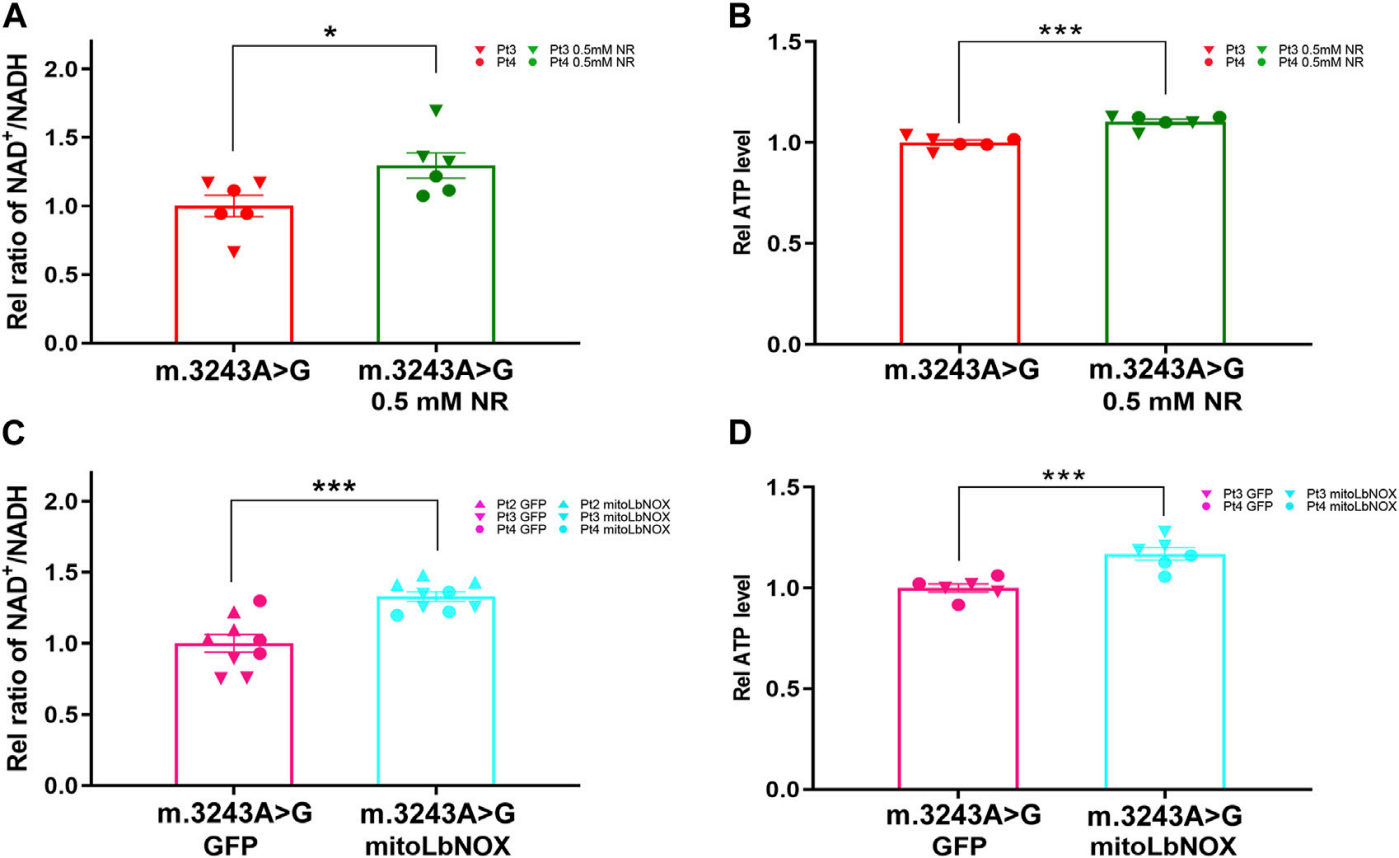
Ratio of NAD^+^/NADH and ATP level were increased in m.3243A>G fibroblasts after treatment with NR and mitoLbNOX. **(A)** Relative NAD^+^/NADH ratio of m.3243A>G fibroblasts treated with 0.5 mM NR compared with untreated m.3243A>G fibroblasts (m.3243A>G n = 6, m.3243A>G 0.5 mM NR n = 6; data were analyzed using a Student’s t-test; error bars represent mean ± SEM; *p < 0.05). **(B)** Relative ATP levels of m.3243A>G fibroblasts treated with 0.5 mM NR compared with untreated m.3243A>G fibroblasts (m.3243A>G n = 6, m.3243A>G 0.5 mM NR n = 6; data were analyzed using a Student’s t-test; error bars represent mean ± SEM; ***p < 0.001). **(C)** Relative NAD^+^/NADH ratio of m.3243A>G fibroblasts transfected with adenovirus expressed GFP or mitoLbNOX (m.3243A>G GFP n = 9, m.3243A>G mitoLbNOX n = 9; data were analyzed using a Student’s t-test; error bars represent mean ± SEM; ***p < 0.001). **(D)** Relative ATP levels of m.3243A>G fibroblasts transfected with adenovirus expressed GFP or mitoLbNOX (m.3243A>G GFP n = 6, m.3243A>G mitoLbNOX n = 6; data were analyzed using a Student’s t-test; error bars represent mean ± SEM; ***p < 0.001). ATP, adenosine triphosphate; GFP, green fluorescent protein; mitoLbNOX, mitochondria-targeted H_2_O-forming NADH oxidase; NAD, nicotinamide adenine dinucleotide; NADH, nicotinamide adenine dinucleotide **+** hydrogen; NR, nicotinamide riboside.

We treated m.3243A>G fibroblasts with either NR or mitoLbNOX (Titov et al., 2016). For NR treatment, m.3243A>G fibroblasts were exposed to 0.5, 1, or 3 mM NR for 7 days (Felici et al., 2015; Douiev and Saada, 2018; Sun et al., 2020). Because 3 mM NR treatment led to high rates of cell death, this treatment group was excluded. We measured the NAD^+^/NADH ratio and ATP levels. The NAD^+^/NADH ratio and ATP levels were elevated in both the 0.5 and 1 mM NR treatment groups, with the 0.5 mM NR treatment group showing a more prominent effect (Supplementary Figure S3). Therefore, 0.5 mM was the optimal dose for NR treatment. After treatment with 0.5 mM NR, the NAD^+^/NADH ratio and ATP level of m.3243A>G fibroblasts were increased by 23% and 10%, respectively, compared to untreated fibroblasts (Figures 3A,B). For enzyme manipulation, we used adenoviral infection to generate m.3243A>G fibroblasts expressing MitoLbNOX (Supplementary Figure S2B), according to a previous report (Titov et al., 2016). In contrast to m.3243A>G fibroblasts expressing only GFP, the NAD^+^/NADH ratio and ATP levels of m.3243A>G fibroblasts equipped with mitoLbNOX increased by 31% and 14%, respectively (Figures 3C,D). Collectively, our results demonstrate that both NR and mitoLbNOX treatment rescued the NAD^+^/NADH ratio, although the effect of mitoLbNOX was more prominent. ATP production was improved more significantly in m.3243A>G fibroblasts expressing mitoLbNOX than in those treated with NR. There was no significant change in m. 3243A>G heteroplasmy levels of m.3243A>G fibroblasts after treating with NR or adenovirus (Supplementary Figure S2C).

### 3.4 Alleviation of NADH accumulation further improved the treatment effect by targeting imbalanced NAD^+^/NADH ratio

**FIGURE 4:**
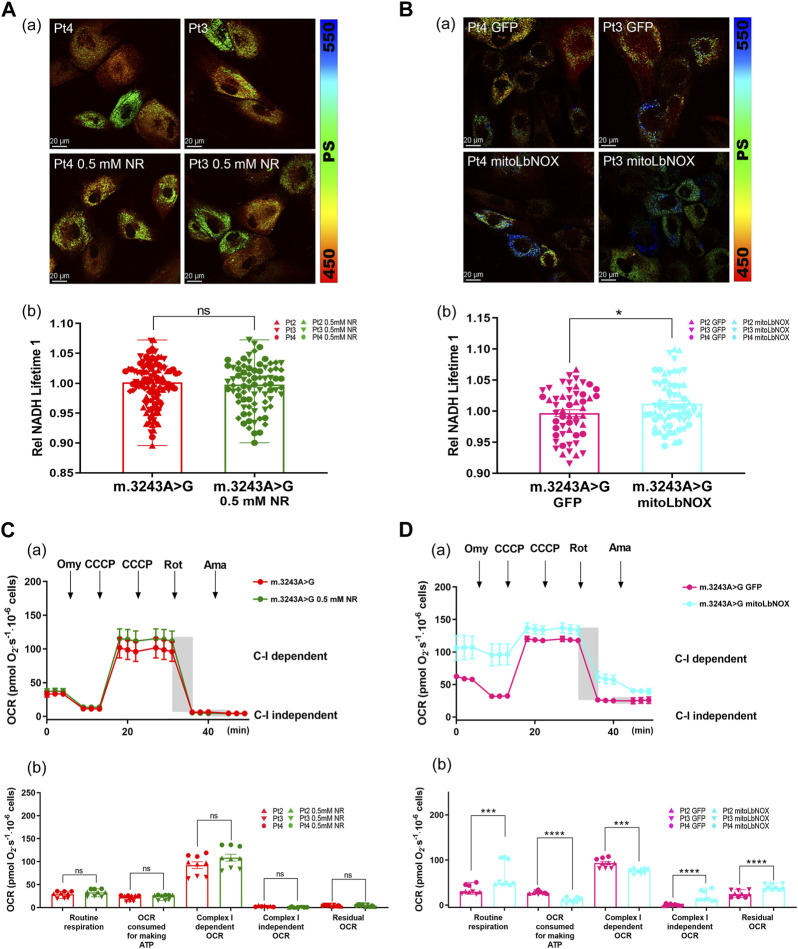
MitoLbNOX exerted a greater effect by oxidizing NADH in m.3243A>G fibroblasts. **(A)** Pseudocolored images **(A)** and quantification **(B)** of relative NADH lifetime in m.3243A>G fibroblasts leaving untreated or treated with 0.5 mM NR (number of fibroblasts counted: m.3243A>G n = 114, m.3243A>G 0.5 mM NR n = 80; data were analyzed using a Mann Whitney test; error bars represent mean ± SEM; ns: not significant). **(B)** Pseudocolored images **(A)** and quantification **(B)** of relative NADH lifetime in m.3243A>G fibroblasts expressed mitoLbNOX or solely GFP (number of fibroblasts counted: m.3243A>G GFP n = 53, m.3243A>G mitoLbNOX n = 65; data were analyzed using a Student’s t-test; error bars represent mean ± SEM; *p < 0.05). **(C)** Oxygraphic measurements **(A)** and analysis **(B)** in m.3243A>G fibroblasts, either untreated or treated with 0.5 mM NR (m.3243A>G n = 3, m.3243A>G 0.5 mM NR n = 3; data were analyzed using a Mann Whitney U test or a Student’s t-test; error bars represent mean ± SEM; ns: not significant). **(D)** Oxygraphic measurements **(A)** and analysis **(B)** in m.3243A>G fibroblasts expressed mitoLbNOX or solely GFP (m.3243A>G GFP n = 9, m.3243A>G mitoLbNOX n = 9; data were analyzed using a Mann Whitney test or a Student’s t-test; error bars represent mean ± SEM; ***p < 0.001, ****p < 0.0001). GFP, green fluorescent protein; MitoLbNOX, mitochondria-targeted H_2_O-forming NADH oxidase; NADH, nicotinamide adenine dinucleotide **+** hydrogen; NR, nicotinamide riboside; SEM, standard error of the mean.

To further investigate the different effects of these two treatments, we used FLIM to evaluate NADH levels in m.3243A>G fibroblasts after treatment with NR or mitoLbNOX. The NADH lifetime had no difference between untreated m.3243A>G fibroblasts and m.3243A>G fibroblasts treated with 0.5 mM NR (Figure 4A). NADH lifetime was significantly increased in m.3243A>G fibroblasts containing mitoLbNOX (Figure 4B), indicating a decrease in free NADH. Taken together, mitoLbNOX more efficiently rescued the NAD^+^/NADH redox balance by oxidizing the excess NADH accumulated in m.3243A>G fibroblasts. We then used the OCR to evaluate the redox transfer of electrons through the, ETC. Although not significantly, m.3243A>G fibroblasts treated with 0.5 mM NR tended to have higher complex I-dependent OCR (Figure 4C; Supplementary Figure S2A). In m.3243A>G fibroblasts infected with an adenovirus expressing mitoLbNOX, the complex I-independent OCR increased by >90% (Figure 4D; Supplementary Figure S2A). The routine respiration of m.3243A>G fibroblasts with mitoLbNOX increased by 48% (Figure 4D), probably due to the enhancement of complex I-independent OCR (Figure 4D).

## 4 Discussion

Since first discovered by Goto et al., 1990 in 1990, m.3243 A>G has accounted for −80% of MELAS patients. To date, targeted treatments (L-arginine, taurine, etc.) and general therapies (antiepileptic treatment, etc.) have been used for treating patients with m.3243A>G related diseases; however, the clinical outcomes are still far from satisfactory (Ng et al., 2021).

Four m.3243A>G fibroblasts with different heteroplasmy levels were used as the cell models in this study. Mitochondrial abnormalities in these fibroblasts included small electron-lucent mitochondria and myelin figures, indicating that autophagic deficiency occurred in the m.3243A>G fibroblasts, which is consistent with previous findings (Deng et al., 2020). The tRNA^Leu(UUR)^ m.3243 A>G mutation affects the translational efficiency of the UUG codon, 42% of which is used by ND6 to decode leucine (Kirino et al., 2004). As indicated in other studies (Sasarman et al., 2008; Karicheva et al., 2011; Homma et al., 2021) and evidenced by our results, complex I is impaired in m.3243A>G fibroblasts, mainly because of the downregulation of ND6 due to the m.3243 A>G mutation. In accordance with previous studies on MELAS cybrid cells (Frey et al., 2017), we demonstrated that complex I deficiency leads to NAD^+^ reduction, NADH accumulation, and decreased ATP production in m.3243A>G fibroblasts.

ATP insufficiency is often considered the principal disturbance of complex I defects, accompanied by oxidative stress, autophagy dysfunction, and other causes (Xiong et al., 2012; Vinogradov and Grivennikova, 2016; Frey et al., 2017). However, crucial evidence has shown that redox balance is disrupted when complex I is impaired, and restoration of this balance is of great importance (Titov et al., 2016; McElroy et al., 2020; Yang et al., 2020). Therefore, this study aimed to evaluate the potential therapeutic effects of restoring the ratio of NAD^+^/NADH in m.3243A>G fibroblasts.

Because the concentration of NADH is higher in mitochondria (Titov et al., 2016), we chose two methods that could particularly elevate the ratio of NAD^+^/NADH in mitochondria to treat m.3243A>G fibroblasts, namely, the addition of NR and the expression of mitoLbNOX. After the intervention, both the ATP level and the ratio of NAD^+^/NADH increased compared to untreated m.3243A>G fibroblasts, which was consistent with previous findings (Majamaa et al., 1996; Jeong et al., 2014; Frey et al., 2017; Seo et al., 2018). Interestingly, mitoLbNOX expression exerted a greater effect than the NR treatment. To further investigate the difference between these two types of interventions, we evaluated NADH lifetime and OCR in m.3243A>G fibroblasts after treatment. For NR applications, complex I-dependent OCR tended to increase, indicating that NAD^+^ may function as an electron acceptor to promote the assembly of complex I, as previously reported (Frey et al., 2017). While there was no difference in NADH lifetime between treated and untreated m.3243A>G fibroblasts, it was unexpected yet reasonable for NR to promote only the biosynthesis of NAD^+^.

**FIGURE 5:**
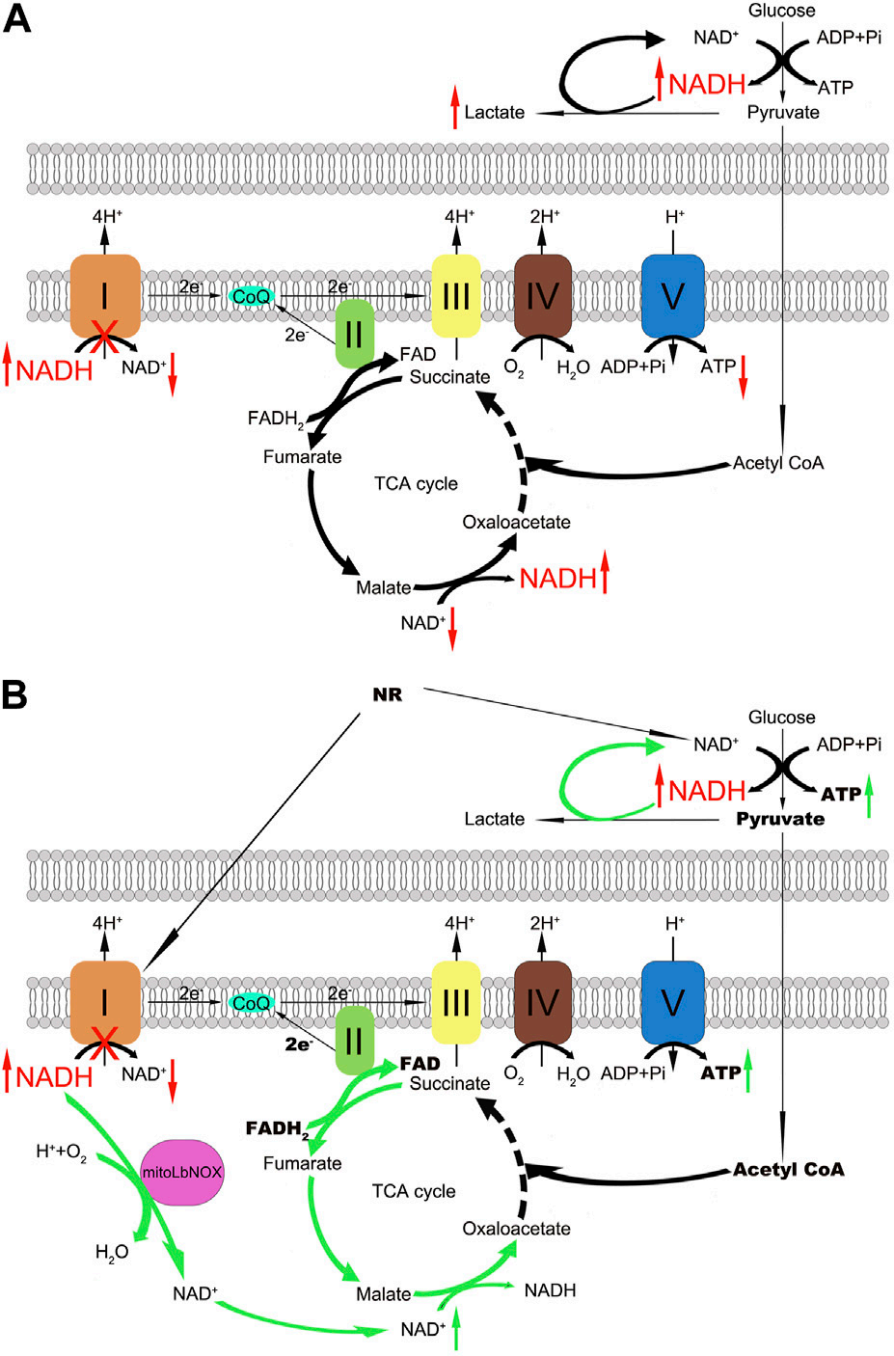
Schematic representation of the respiratory chain and the effects of NR and mitoLbNOX in m.3243A>G fibroblasts. **(A)** Complex I oxidizes NADH and regenerates NAD^+^, dysfunction of complex I leads to NADH accumulation and NAD^+^ depletion. **(B)** NR treatment and mitoLbNOX expression replenish m.3243A>G fibroblasts with NAD^+^, and mitoLbNOX exerts a greater effect by consuming the excess NADH accumulated in cells. MitoLbNOX, mitochondria-targeted H_2_O-forming NADH oxidase; NAD, nicotinamide adenine dinucleotide; NADH, nicotinamide adenine dinucleotide + hydrogen; NR, nicotinamide riboside.

MitoLbNOX replenishes NAD^+^ levels in cells by oxidizing NADH and directly transferring electrons to oxygen (Titov et al., 2016). Two factors lead to decreased NAD^+^/NADH ratio: NAD^+^ depletion and NADH accumulation. In contrast to the extensive investigation of NAD^+^ depletion, few studies have focused on the role of NADH accumulation. Several recent findings in Leigh syndrome models indicate that NADH accumulates in cells, leading to impaired oxidative metabolism due to complex I deficiency (McElroy et al., 2020; Yang et al., 2020). Thus, we inferred that mitoLbNOX exerts a greater effect by consuming the excess NADH accumulated in m.3243A>G fibroblasts. Our results support this hypothesis; particularly the increased lifetime of NADH in m.3243A>G fibroblasts treated with mitoLbNOX. It is noteworthy that complex I-independent OCR in m.3243A>G fibroblasts transfected with mitoLbNOX increased significantly, indicating that mitoLbNOX facilitated the activation of the TCA cycle after the alleviation of NADH overload, thus promoting electron transfer through complex II and having a greater treatment effect (Figure 5).

There were several limitations in the present study. Due to proliferation defects and discrepancy in growth, not all fibroblast lines were included in all the experiments. The redox ratio is important in culture settings, and the role of complex I deficiency or redox imbalance in the pathology of MELAS should be further investigated *in vivo*.

## 5 Conclusion

In conclusion, our results indicate that NADH overload contributes to the pathogenesis of m.3243A>G mutation. In addition to NAD^+^ repletion, alleviation of excess NADH in m.3243A>G fibroblasts makes strategies targeting an imbalanced NAD^+^/NADH ratio more effective. Hence, combining alleviation of NADH accumulation with NAD^+^ repletion may be a promising treatment strategy for patients with m.3243A>G mutation.

## Data Availability

The original contributions presented in the study are included in the article/Supplementary Material, further inquiries can be directed to the corresponding authors.

## Supplementary material

SUPPLEMENTARY FIGURE S1
**(A)** The m.3243A>G mutation of m.3243A>G fibroblasts were confirmed by Sanger sequencing. **(B)** The range of heteroplasmy for each m.3243A>G fibroblast line at the relative passages (p) when the experiments were taken was determined by RFLP analysis compared to negative control and positive control. **(C)** Quantification of the complex IV activity in m.3243A>G and control fibroblasts (Pt2 n = 3, Pt3 n = 3, Pt4 n = 3, Pt5 n = 3, Control n = 6; data were analyzed using a Student’s t test; error bars represent mean ± SEM; **p* < 0.05, ****p* < 0.001). Pt, patient; RFLP, Restriction fragment length polymorphism; SEM, standard error of the mean.

SUPPLEMENTARY FIGURE S2
**(A)** Original representative OCR graphs in different groups. **(B)** Western blot analysis of Flag expression in m.3243A>G fibroblasts treated with adenovirus expressed GFP or mitoLbNOX. **(C)** RFLP analysis in fibroblasts of Pt4, either untreated or treated with 0.5 mM NR or treated with adenovirus expressed GFP or mitoLbNOX. GFP, green fluorescent protein; MitoLbNOX, mitochondria-targeted H_2_O-forming NADH oxidase; OCR, oxygen consumption rate; Pt, patient; RFLP, Restriction fragment length polymorphism.

SUPPLEMENTARY FIGURE S3 Effect of 1 mM NR on m.3243A>G fibroblasts. **(A)** Relative NAD^+^/NADH ratio of m.3243A>G fibroblasts treated with 1 mM NR compared with untreated m.3243A>G fibroblasts (m.3243A>G n = 6, m.3243A>G 1 mM NR n = 6; data were analyzed using a Student’s t test; error bars represent mean ± SEM; **P* < 0.05). **(B)** Relative ATP levels of m.3243A>G fibroblasts treated with 1 mM NR compared with untreated m.3243A>G fibroblasts (m.3243A>G n = 9, m.3243A>G 1 mM NR n = 9; data were analyzed using a Student’s t test; error bars represent mean ± SEM; **P* < 0.05). **(C)** Pseudocolored images (a) and quantification (b) of relative NADH lifetime in m.3243A>G fibroblasts leaving untreated or treated with 1 mM NR (number of fibroblasts counted: m.3243A>G n = 44, m.3243A>G 1 mM NR n = 40; data were analyzed using a Student’s t test; error bars represent mean ± SEM; ns: not significant). (D) Oxygraphic measurements (a) and analysis (b) in m.3243A>G fibroblasts, either untreated or treated with 1 mM NR (m.3243A>G n = 3, m.3243A>G 1 mM NR n = 3; data were analyzed using a Mann Whitney test or a Student’s t test; error bars represent mean ± SEM; ns: not significant). ATP, adenosine triphosphate; GFP, green fluorescent protein; mitoLbNOX, mitochondria-targeted H_2_O-forming NADH oxidase; NAD, nicotinamide adenine dinucleotide; NADH, nicotinamide adenine dinucleotide **+** hydrogen; NR, nicotinamide riboside; SEM, standard error of the mean.
