# Supplementary figures and images for: Complex I deficiency in m.3243A>G fibroblasts is alleviated by reducing NADH accumulation
Source: Front Physiol. 2023 Aug 15;14:1164287. doi: 10.3389/fphys.2023.1164287 (PMC10464909; doi:10.3389/fphys.2023.1164287)

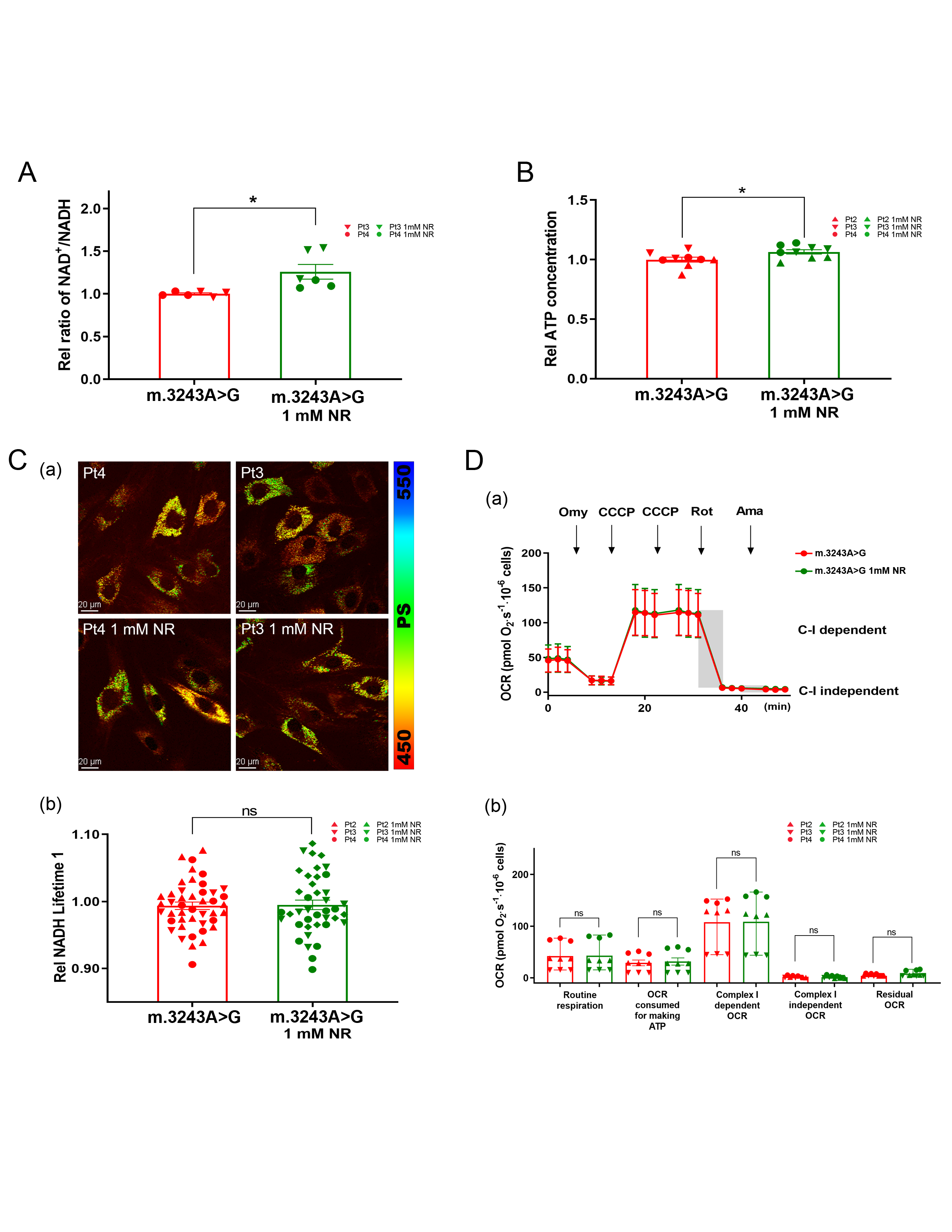

Supplement: Supplementary file 1 [file Image3.tif]

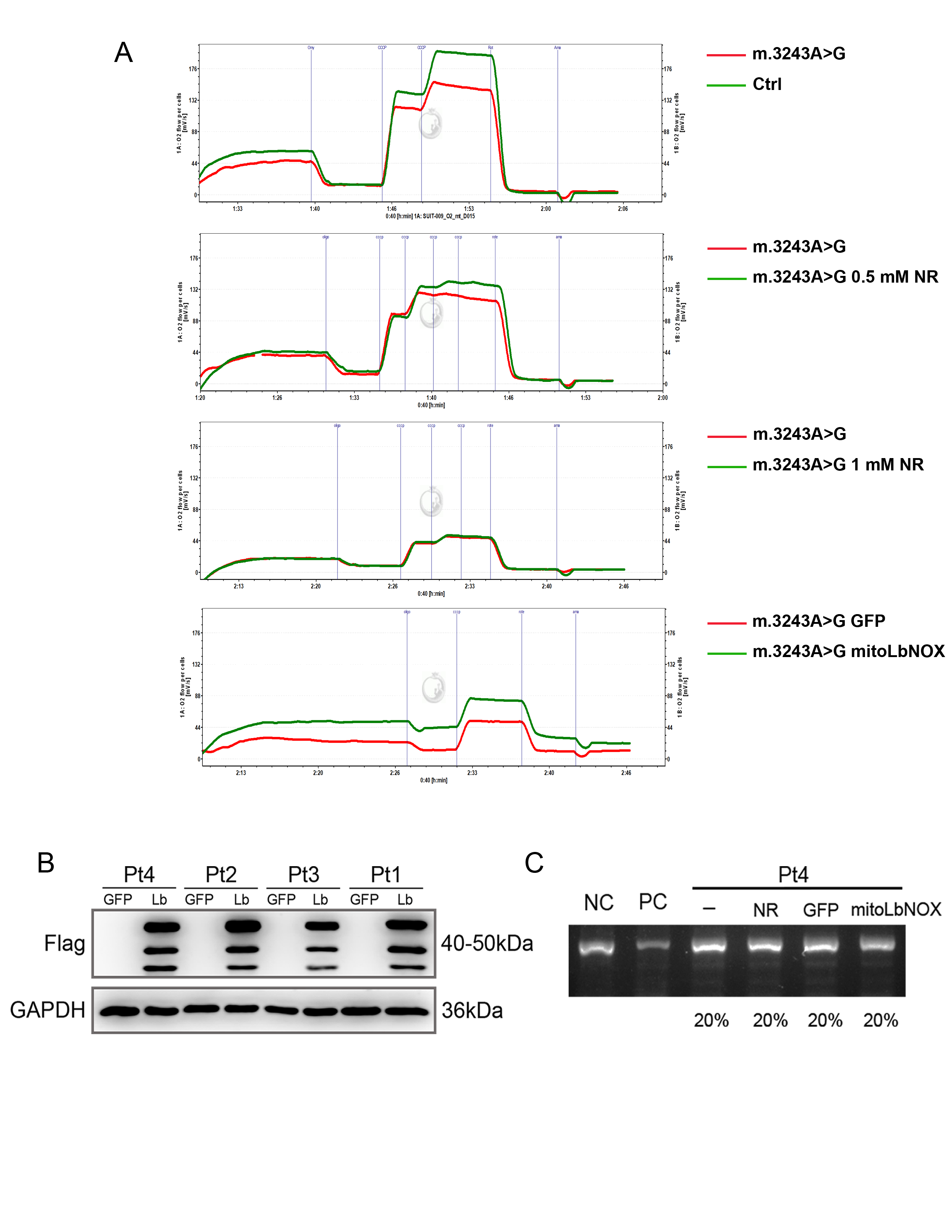

Supplement: Supplementary file 2 [file Image2.tif]

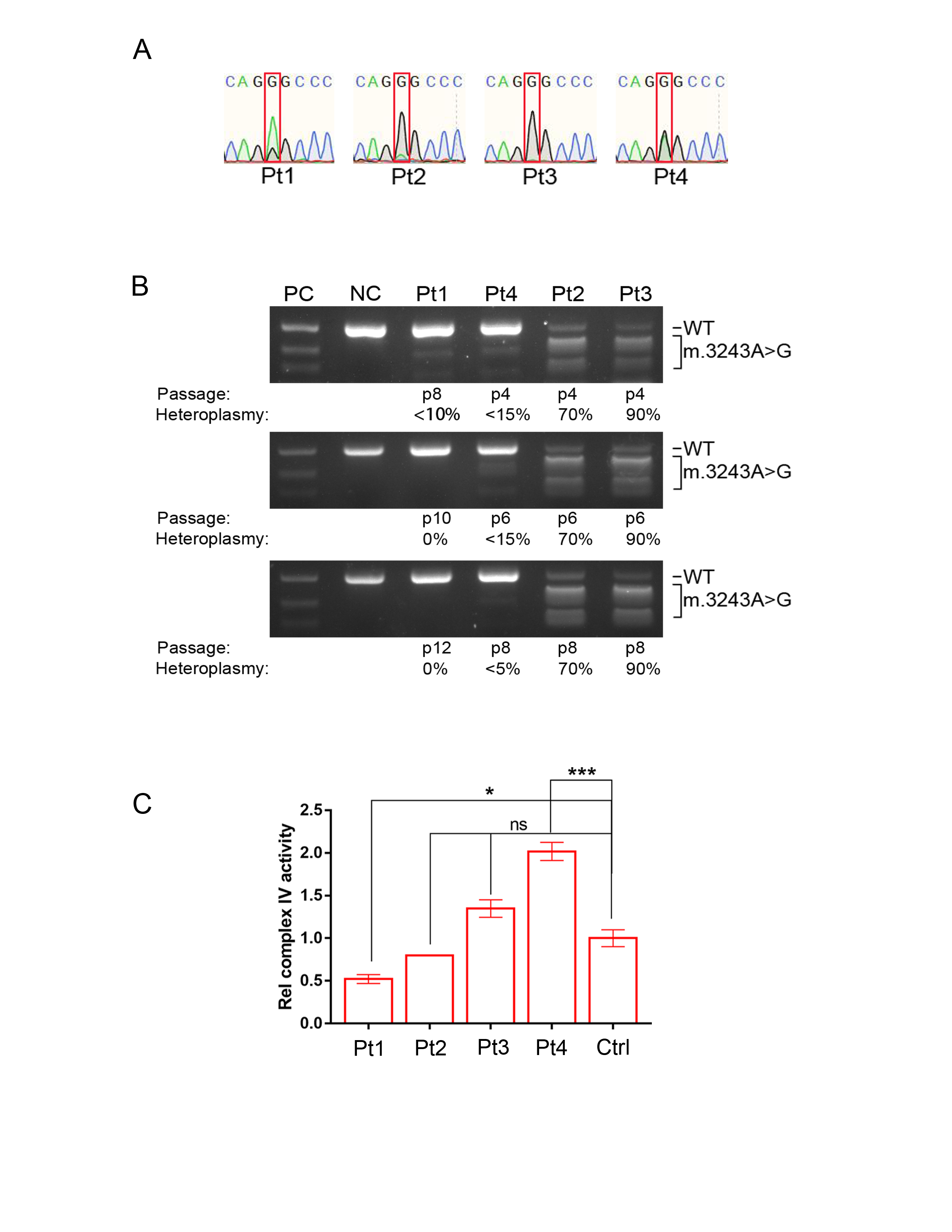

Supplement: Supplementary file 3 [file Image1.tif]
